# Validating the application of cyclic hydraulic pressure pulses to reduce breakdown pressure in granite

**DOI:** 10.1016/j.isci.2024.110881

**Published:** 2024-09-03

**Authors:** Jackie Evan Kendrick, Anthony Lamur, Julien Mouli-Castillo, Alexander Lightbody, Andrew Fraser-Harris, Katriona Edlmann, Christopher Ian McDermott, Zoe Kai Shipton

**Affiliations:** 1 Department of Earth and Environmental Sciences, Ludwig Maximilian Universität 80333 Munich, Germany; 2 School of Geosciences, University of Edinburgh, Edinburgh EH9 3FE, UK; 3 James Watt School of Engineering, University of Glasgow, Glasgow G12 8QQ, UK; 4 Energy Technologies Area, Lawrence Berkeley National Laboratory (LBNL), Berkeley, CA, USA; 5 Department of Civil and Environmental Engineering, University of Strathclyde, Glasgow G1 1XJ, UK

**Keywords:** Earth sciences, Geology, Applied geology, Methods in earth sciences, Petrophysics

## Abstract

As the geoenergy sector moves toward more sustainable practices, an emerging field of research is the proposed utilization of cyclic hydraulic pressure pulses to safely and efficiently enhance productivity. We demonstrate how cyclic hydraulic pressure pulses can reduce hydraulic breakdown pressure in granite using newly developed experimental equipment, which applies pulsed square waves of fluid pressure to large bench-top samples, monitored with dynamic high-resolution fiber optic strain sensors. Our results show a significant reduction in breakdown pressure can be achieved by cyclic pulsed pumping, and we explore the role of mean pressure and cyclic amplitude. Our results offer new insight into cyclic well-stimulation treatments and show potential for reducing peak power consumption during geothermal exploitation.

## Highlights

• Hydraulic breakdown pressure in granites is reduced using cyclic pressure pulses
• Lower pressure at failure implies reduction in size of the largest stress-drop events
• Cyclic pressure pulsing may reduce peak power consumption for geo-energy stimulation

## Graphical abstract

**Figure:**
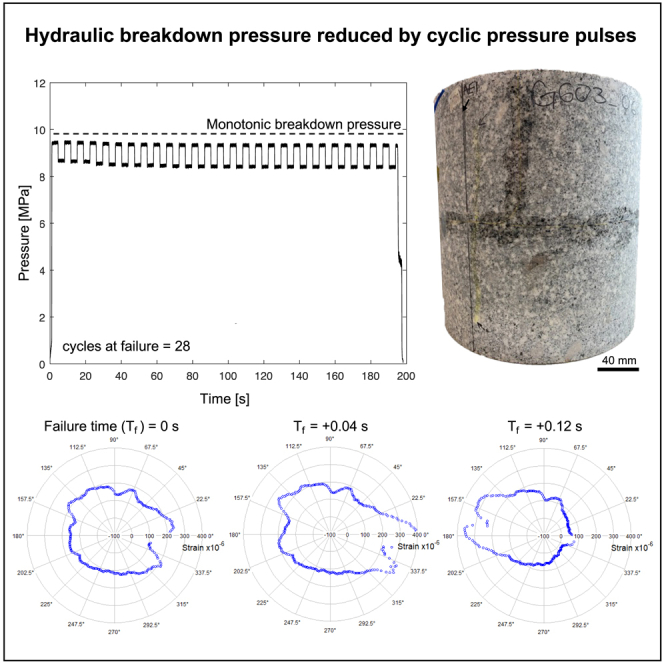

## Introduction

Low carbon geoenergy systems are an integral part of the global shift toward more sustainable economies. Key to these systems is a comprehensive understanding of subsurface structures and how to access and manipulate them safely and efficiently. In particular, geothermal energy seeks to maximize energy extraction and ensure the longevity of the heat resource, while minimizing induced and triggered seismicity and avoiding the risk of environmental contamination.^1^ Faults and fractures in upper crustal rocks used for geothermal production provide both the opportunity and the challenge.^2^ On the one hand fractured rocks allow the efficient percolation of fluids and provide large surface areas for energy exchange between rocks and fluid,^3^ on the other, even small shifts in pore fluid pressure can instigate fault slip, and fractures can grow and coalesce unchecked.^4^^,^^5^ A plethora of experimental and theoretical work has focused on the maximization of geothermal resources. In particular, in recent years cyclic hydraulic pressure pulse treatment has been proposed as a means to enhance near-well permeability^6^^,^^7^^,^^8^^,^^9^^,^^10^ whilst providing improved control of the well stimulation process.^11^

Experimental efforts have demonstrated that when implementing cyclic hydraulic pressure pulses, permeability can be increased, and fracture propagation controlled, reducing the occurrence of microseismic events compared to monotonic stimulation.^4^^,^^7^^,^^12^ In traditional monotonic stimulation practices, constant flow rates or pressurization rates are applied until overpressure exceeds the rock strength or cohesion of an existing fracture/fault, at which point a rupture forms or propagates, and a stress drop ensues, releasing seismicity.^4^^,^^13^ Although the total seismic energy released by fracturing may be equivalent when using cyclic hydraulic pressure pulses, the proportion of large events can be reduced by the instigation of frequent small events, serving to reduce seismic *b*-value.^14^ The associated small fracturing events represent staged fracture growth,^15^ described by the Paris-Erdogan law^16^ and can produce complex fracture networks^7^^,^^9^ that efficiently sustain permeability for protracted energy extraction.^17^ Experimental results have recently been validated by early field tests which demonstrated the relatively small maximum magnitude of induced seismic events.^18^

Despite the relatively recent uptake of cyclic exploitation practices in geothermal, and more broadly, geoenergy and exploration industries, the approach echoes the findings of decades of work related to fatigue and cyclic loading of rocks and other geomaterials,^19^ which demonstrate the progressive fatigue by sub-critical crack growth due to distributed microfracturing and decohesion of the rock structure.^20^ Field-tests have demonstrated the feasibility of applying cyclical pressure pulses in geothermal settings^18^ and when optimized, the implementation of cyclic hydraulic pressure pulse techniques have the potential to reduce peak energy consumption.^21^ Now, as a community we must find a solution to maximize our conceptual understanding of fatigue to implement efficient and safe exploitation strategies^4^ within the framework of what is technically achievable.

Here, we explore how the peak pressure and cycle amplitude impact breakdown pressure in large-scale granite samples (Ø 200 mm, and length 200 mm) using a novel experimental setup. Our setup allows the pump to operate at a constant flow rate and pressure, whilst delivering a square wave of set frequency (here 0.14 Hz) to the rock sample by shifting from a high to low pressure line. We control the mean pressure and cycle amplitude of the square wave and monitor the sample with fiber optic strain measurements at high-resolution (every 2.6 mm around the circumference of the sample) at 25 Hz frequency. The results of 2 monotonic and 10 cyclic loading tests explore the effects of applying a new type of pressure wave to real rock samples for the first time. The resultant reduction in breakdown pressure is assessed in the context of other studies on cyclic loading, and implications for utilizing square wave cyclic pulsing in geothermal exploitation are discussed.

## Results

### Monotonic breakdown pressure

**Figure 1:**
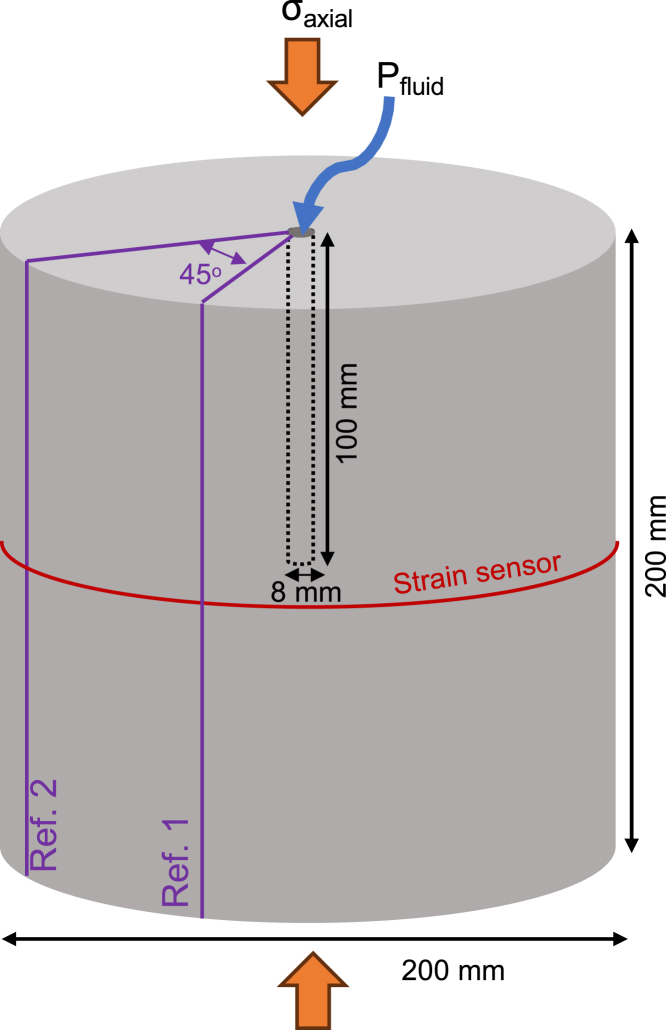
Sample geometry and dimensions for monotonic and cyclic hydraulic fracture tests The 200 × 200 mm cylindrical sample was held at an axial stress (σ_axial_) of 8 MPa and water was pumped into the 8 mm central borehole as pressure (P_fluid_) and radial strain were monitored. Two markers (ref. 1 and ref. 2) were used for spatial referencing of the fiber optic cable around the circumference of the sample.

**Figure 2:**
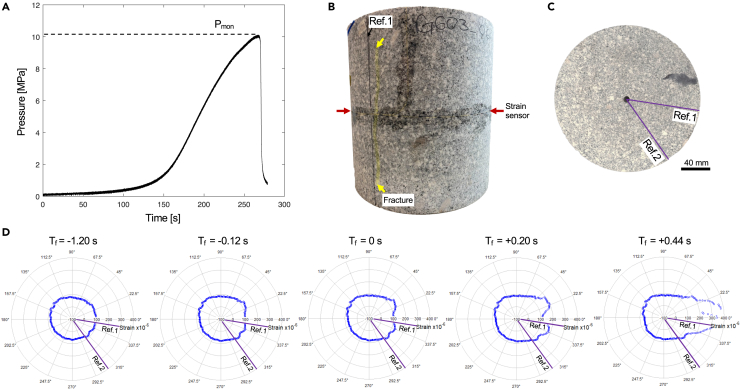
Monotonic hydralic fracture experiment: pressure evolution, fracture and strain evolution A monotonic hydraulic fracture test, showing (A) The pressure log through time during constant flow rate pressurization, resulting in a hydraulic fracture used to define the monotonic breakdown pressure, P_mon_ (B) A side view of the sample following testing, exhibiting a vertically extended fracture which cross-cuts the fiber-optic strain sensor around the center of the sample. (C) A top-down view of the sample following testing. No fracture trace is visible on the top surface, but reference lines 1 and 2 for angular positioning of the strain sensor are indicated. (D) A series of time steps showing the strain around the circumference of the sample before, during, and after the time of failure (T_f_). The orientation of the sample in (C) and strain data in (D) are the same.

Two samples of G603 granite were subjected to unconfined monotonic tests in which a constant flow rate increased the water pressure in the central borehole (Figure 1) until a hydraulic fracture formed, connected to the outer margin of the sample and the fluid pressure was released. In the two experiments, the breakdown pressure was found to be 9.63 MPa and 10.11 MPa (Figure 2A, and supplemental information), providing an average of 9.87 MPa. The slight difference can be attributed to the visibly heterogeneous nature of these granites, which contain large feldspar phenocrysts (typically 1 mm up to 8 mm, and occasionally up to 20 mm) sparsely and heterogeneously distributed within the sample (Figures 2B and 2C). The remaining mineralogical assemblage is more homogeneous, consisting of quartz and plagioclase (typically 0.5–1.5 mm) and mica (individually <0.5 mm). A previous study on samples from the same blocks by Kendrick et al.^22^ also found minor variation in the porosity, which averaged 1.00% (S.D. 0.13) and density, averaging 2.63 g cm^−1^ (S.D. 0.01), though our 200 × 200 mm cylinders were too large to characterize individually.

Despite the small difference in the monotonic breakdown pressures for the two experiments, they behaved very similarly. During the monotonic tests, the flow rate applied was constant, and the fluid pressure within the borehole increased slowly and non-linearly initially, until approximately 2 MPa (approximately 20% of the ultimate breakdown pressure). From 2 MPa until approximately 8 MPa the pressure increased linearly for about 1 min (Figure 2A), which can be considered as the elastic portion of deformation of the rock specimen. Beyond this point (8 MPa, ∼80% of the ultimate breakdown pressure) the rate of pressure increase slowed, attributed to the onset of damage accumulation. The onset of damage accumulation can be seen in the fiber optic strain data in the fractions of a second prior to failure (failure time, T_f_ = −0.12 s), in which a small double bulge, which indicates sample dilatancy, appears in the strain measurements around the circumference of the sample (at approximately 030° and 320° in Figure 2D). These bulges flank the hydraulic fracture that ultimately forms (centered at 005° in Figure 2D, T_f_ = 0 s). When the fracture forms it connects the pressurized fluid to the outside of the sample, releasing the pressure near-instantly (Figure 2A). We see evidence for only a single fracture, which spans almost the complete vertical length of the sample but does not intersect the upper or lower surface (Figures 2B and 2C), which corroborates the strain data (Figure 2D) that shows dilatancy localized to the quadrant spanning the fracture, and growing rapidly in the fractions of a second following failure.

In our unconfined geometry the minimum principal stress is 0 MPa, so for a hydraulic fracture to propagate it must only exceed the tensile strength of the material.^23^ The average monotonic breakdown pressure (P_mon_) of 9.87 MPa is close to the 9.61 MPa tensile strength measured for the same material at an average stress accumulation rate of 0.2 MPa/s,^22^ similar to the 0.1 MPa/s accumulated during the linear pressure increase in the tests here.

### The impact of cyclic hydraulic pressurization on fracturing

**Figure 3:**
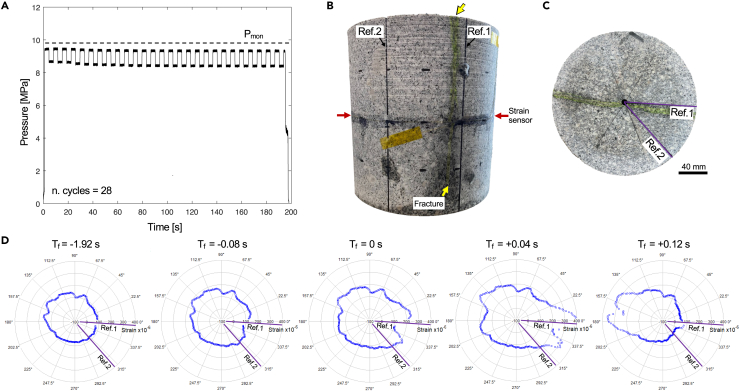
Cyclic hydralic fracture experiment: pressure evolution, fracture and strain evolution A cyclic hydraulic fracture test, showing (A) The pressure log through time during pressure pulse cycles, here with a cycle amplitude of 10% of P_mon_ with P_max_ set at 95% of P_mon_ (hence a P_mean_ of 90%). The data are compared to the average P_mon_ 9.87 MPa. (B) A side view of the sample following testing, exhibiting a fracture extending from top to bottom, and which cross-cuts the fiber-optic strain sensor around the center of the sample. (C) A top-down view of the sample following testing showing a fracture extending laterally in both directions from the central borehole (highlighted in green). Reference lines 1 and 2 for angular positioning of the strain sensor are indicated. (D) A series of time steps showing the radial strain before, during, and after the time of failure (T_f_). The orientation of the sample in (C) and strain data in (D) are the same.

Ten samples of G603 granite were subjected to unconfined cyclic pulsed pumping tests in which 0.14 Hz frequency square pressure waves were applied via water pressure in the central borehole (Figure 1; after Mouli-Castillo et al; ^15^). Mean, maximum and minimum pressures (P_mean_, P_max,_ and P_min_, respectively) and amplitudes of the cycles were defined as a fraction of the monotonic breakdown pressure (P_mon_; Table 1). In each case, the high pressure-low pressure cycles alternated until a fluid pressure drop occurred (Figure 3A), caused by the propagation of a hydraulic fracture from the pressurized central borehole out to the sample margin (Figures 3B and 3C). In all cases, we found that the hydraulic fracture was initiated during the high-pressure step.

**Table 1:** Experimental parameters for the cyclic tests with P_mean_, P_max_, P_min,_ and amplitude of the cycles in MPa and as % of P_mon_, along with calculated pressure ratio and the resulting number of cycles to failure

P_mean_ [MPa]	P_mean_ [% P_mon_]	P_max_ [MPa]	P_max_ [% P_mon_]	P_min_ [MPa]	P_min_ [% P_mon_]	Cycle amplitude [MPa]	Cycle amplitude [% P_mon_]	Pressure ratio	No. of cycles to failure
5.9	60	6.9	70	4.9	50	2.0	20	0.70	>1000^a^
7.4	75	8.9	90	5.9	60	3.0	20	0.90	936
7.4	75	9.4	95	5.4	55	4.0	30	0.95	53
7.4	75	8.4	85	6.4	65	2.0	41	0.85	241
7.9	80	8.9	90	6.9	70	2.0	30	0.90	107
7.9	80	9.4	95	6.4	65	3.0	20	0.95	1
7.9	80	8.4	85	7.4	75	1.0	10	0.85	214
8.4	85	9.4	95	7.4	75	2.0	10	0.95	2
8.4	85	8.9	90	7.9	80	1.0	20	0.90	232
8.9	90	9.4	95	8.4	85	1.0	20	0.95	28

a Indicates no failure occurred.

Similarly to the monotonic tests, damage accumulation can be seen in the fiber optic strain data in the moments before failure. Dilatancy is observed via a double bulge in the radial strain measurements (at approximately 010° and 315° in Figure 3D) which flanks the soon-to-initiate hydraulic fracture (centered at 350° in Figure 3D, T_f_ = 0 s). As the fracture propagates to the sample margin in the fractions of a second following (T_f_ = +0.04 s), the dilatancy increases, but maintains the double bulge geometry surrounding the fracture. Shortly afterward, a pronounced expansion with the same characteristic shape is seen on the opposite side to the initial fracture (centered at 165° in Figure 3D, T_f_ = +0.12 s). Indeed, in all the pulsed pumping cases we saw that the fracture propagated from the borehole to the sample margin (Figure 3B) first in a single direction, and then, propagated back: from the borehole to the opposite side (Figure 3C), creating a fracture plane that dissected all but the base of the cylindrical samples. We attribute this to the constant supply of pressurized fluid even after fracture initiation (due to the bladder accumulator which buffers against pressure drops and supplies constant pressure even in the event of large system volume increases) which served to maintain fracture growth. This is in contrast to the monotonic case in which a constant flow rate supplied the pressurizing fluid, and thus the pressure is lost as the fracture increases the system volume, an effect verified by numerical modeling of hydraulic fracture initiation.^24^ As such, the back-propagation of the fracture from the borehole to the opposite margin of the sample following the first hydraulic fracture in our pulsed pressure experiments is considered to be an artifact of our experimental procedure because the bladder accumulator volume is orders of magnitude higher than our system volume, which is unrealistic for natural scenarios. As such it is unlikely to accurately represent the post-fracturing behavior of a natural system subjected to fluid pressure pulses, in which the system would likely lose pressure rapidly at fracture initiation before building again, leading to a second phase of fracture growth.^24^ Indeed, in similar experiments using square pressure pulses applied without the bladder accumulator, Mouli-Castillo et al.^15^ showed staged fracture growth.

### The impact of maximum pressure during cyclic hydraulic pressurization

For all tests, sample failure was indicated by a drop in fluid pressure. For cyclic tests, the number of cycles that occurred prior to failure was recorded (Table 1). It is typically considered that the peak pressure of cycles has the dominant control on the number of cycles to failure, but traditionally the role of mean pressure and cycle amplitude have been somewhat overlooked. Here, we explore P_mean_, P_max_, P_min,_ and amplitude of the cycles, cast in terms of P_mon_, and how they impact the number of cycles to failure.

We note that all but one sample failed before our end threshold of 1000 cycles. The remaining experiments failed during the high-pressure portion of the cycle, thus P_fail_ = P_max_ and so we calculate the pressure ratio as P_max_/P_mon_, which can be compared to a number of cycles to failure, which is known as the fatigue life of the granite.

**Figure 4:**
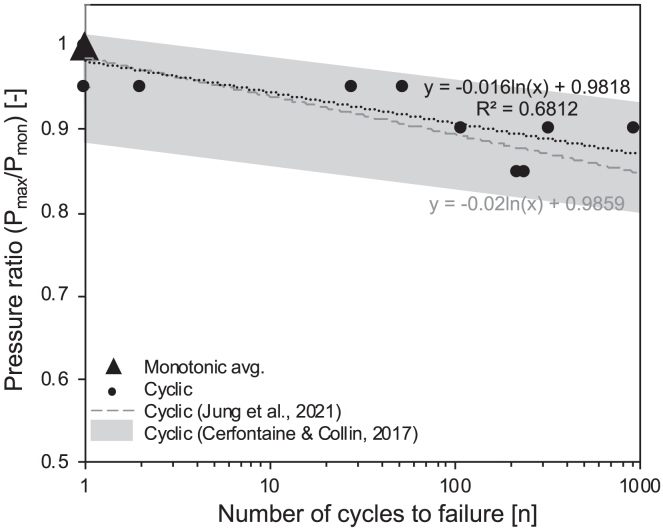
The fatigue lifespan of the granite samples An S-N plot showing the pressure ratio (P_max_/P_mon_) and the resulting number of cycles to failure for all tests. Monotonic breakdown can be indicated on the plot at point 1,1 since by definition a monotonic test fails at P_mon_ during its first and only cycle, here the average monotonic breakdown pressure occupies this point. For reference we plot the two individual monotonic tests (which comprise the average) with the cyclic data, taking P_max_ as their individually recorded breakdown pressures and P_mon_ as the average monotonic breakdown pressure to define their “apparent” pressure ratios. Their position provides a proxy for sample variability. We find that the number of cycles to failure increases exponentially with decreasing pressure ratio. The data are compared to data for granites in cyclic hydraulic loading tests from Jung et al.^10^ and to the regime defined by Cerfontaine and Collin^19^ for cyclic and fatigue testing of rocks in a broad suite of deformation regimes. The regression provided for our data has an R^2^ value of 0.68, whilst that of Jung et al.^10^ has an R^2^ of 0.58.

The experiments show that the higher the pressure ratio, the lower the number of cycles to failure (Figure 4). In other words, the fatigue life of our samples increased exponentially with decreasing P_max_ until the pre-defined experimental limit of 1000 cycles. We see that failure can result from a pressure ratio of as low as 0.85, a 15% reduction in the peak pressure compared to the monotonic case (even with our limit of 1000 cycles). This compares well to other experimental studies showing reductions of up to 20%^10^^,^^25^ and modeled reduction in breakdown pressure of 10–18%^24^ using hydraulic pulses. We note a high variability in the number of cycles to failure with the same P_max_ (Figure 4), but this results from the deliberate variation of P_mean_ (hence cycle amplitude and P_min_) which is not included in this figure and will be explored in the next section. We can define the regression for fatigue life or number of cycles to failure (n) as a function of pressure ratio (P_max_/P_mon_) in our experiments:

(Equation 1) n=exp[(PmaxPmon)−0.9818−0.016]

Despite our systematic variation of cyclic amplitude, we see that the relationship is highly comparable to other cyclic hydraulic pressurization experiments that used a repeating pressure pulse dissipating to near ambient pressure at variable frequencies in granites.^10^ More broadly we see good agreement with the regime defined by Cerfontaine and Collin^19^ for fatigue and cyclic testing on a broad range of geomaterials (including marble, granite, sandstone, and tuff) in tension, compression and triaxial settings (Figure 4).

### The impact of mean pressure and amplitude during cyclic hydraulic pressurization

**Figure 5:**
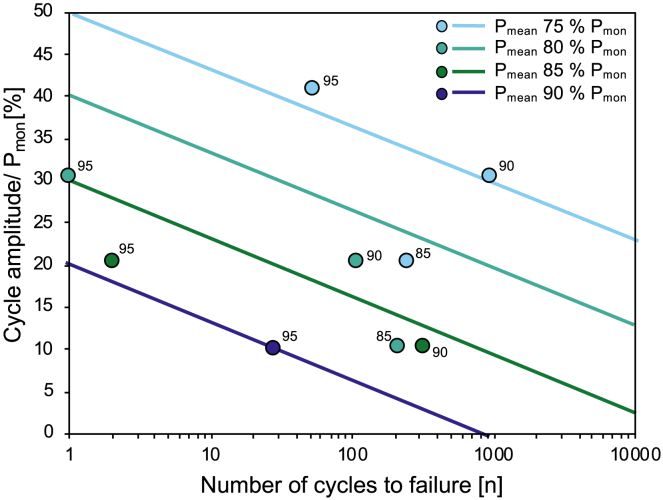
Cycle amplitude as a % of the monotonic breakdown pressure P_mon_ versus number of cycles to failure for different mean borehole fluid pressures (indicated in the key) Data labels on individual points represent P_max_ as a % of P_mon_. To capture the overall effect of the experimental variables, the regressions were defined by fitting a relationship for each P_mean_ (see Figure S11), the exponents were then averaged to define a common slope to describe the population, and plotted with an intercept on the y axis (at *n* = 1 on the x axis) prescribed such that P_max_ equals 100% P_mon_ for a given P_mean_ (e.g., 80% P_mon_) and amplitude (e.g., 40% P_mon_), which by definition should fail on cycle 1.

The role of P_mean_ and cycle amplitude were investigated by systematically varying them whilst maintaining defined values of P_max_ (95, 90, 85% of the monotonic breakdown pressure P_mon_). Four P_mean_ conditions were tested: 75, 80, 85, and 90% of the monotonic breakdown pressure P_mon_. Four cycle amplitudes were tested: 10, 20, 30, and 40% of P_mon_. The results can be framed in terms of the cycle amplitude versus the number of cycles to failure, while distinguishing the experiments by their P_mean_ (Figure 5). Here we consider only the 9 tests that resulted in sample failure.

We find that, for a given P_mean_ (e.g., P_mean_ 80% of P_mon_, Figure 5), reducing cycle amplitude increases the number of cycles to failure, as this has the effect of reducing P_max_. We also see that for a given cycle amplitude (e.g., 20% of P_mon_, Figure 5), reducing the P_mean_ increases the number of cycles to failure, again, as this reduces P_max_. We can also use this plot to predict the number of cycles to failure for any condition of P_max_, P_mean,_ and cycle amplitude. What this reinforces is that P_max_ has the dominant control on fatigue life, but it highlights that even small pressure cycle amplitudes can be effective in inducting fatigue-driven hydraulic fracture propagation.

## Discussion

In this section, we will discuss our findings in the context of geo-energy applications. The average breakdown pressure in the monotonic hydraulic fracturing tests of 9.87 MPa (9.63 MPa and 10.11 MPa) is close to the 9.61 MPa tensile strength,^22^ showing promise for the extrapolation of tensile strength measurements to the pressure needed to perform hydraulic stimulation of *in situ* rock. In a confined setting, theory dictates that one should simply have to consider the minimum principal stress (often overburden/depth), which can be added to the tensile strength following^23^^,^^26^ to define the overpressure required for hydraulic fracturing. A recent study of hydraulic fracturing of PMMA provided experimental validation of this theory as the same fluid overpressure (the difference between fluid pressure and confining pressure) was required to induce a fracture under confined and unconfined conditions.^15^ However, further experimental validation on natural heterogeneous geomaterials, as well as in situin-situ (subsurface) verification, is required.

Our results show that the maximum pressure of the applied pressure pulses has the dominant control on the fatigue life of the examined low-porosity granites. We see a reduction in peak pressure required for sample failure of up to 15% compared to monotonic breakdown (in other words, we can induce failure at 85% of monotonic breakdown pressure) even considering our pulse limit of 1000 cycles (∼2 h at the tested frequency). Considering that for pumps, energy consumption is highest when the highest fluid pressures are demanded, then a reduction in necessary peak pressure should lower the peak energy consumption of equipment utilized for geothermal energy extraction.^27^ We surmise that our observations are likely to be repeatable in other rock types and stress settings: fatigue life has been previously observed to be equivalent for a broad range of lithologies in a spectrum of stress fields and geometries, and most geomaterials have comparable reductions in peak pressure (or stress) required to induce failure during cyclic loading/pressurisation.^9^^,^^14^^,^^19^^,^^28^ This observation remains, despite the application of different cycle frequencies, amplitudes, and geometries (sinusoidal, square, pulse-decay, and so forth) and can be attributed to the irreversible accumulation of strain due to microfracturing and decohesion of the rock structure under prolonged stressing or stress cycling.^20^^,^^29^ Within this spectrum, minimum pressure has rarely been explored, yet may be a vital parameter to be understood in geothermal prospecting where the fluid-saturated environment (elevated pore pressure) will dictate P_min_. Our experiments have systematically explored the impact of varying the mean, maximum and minimum pressures, and amplitude of the pressure pulses in the wellbore. We show that pulse amplitudes of 10% of the monotonic breakdown pressure can induce fatigue in the granites (depending on the P_mean_). Therefore, although P_max_ has the dominant control on fatigue life, even small pressure fluctuations, which may be relatively easily implemented during geoenergy extraction efforts, can be effective in inducing fatigue-driven hydraulic fracture propagation.

We use fiber optic strain measurements to track the sample during cyclic pressurization and the induced hydraulic fracturing events. We identify an increase in strain (i.e., dilation) in the adjacent quadrant to the eventual fracture in the fractions of a second prior to failure. The identification of dilatancy during hydraulic stimulation prior to fracturing may be valuable in providing a way to monitor fracture initiation and provide the opportunity to develop control systems to tune fracture growth and even halt operations if fracture growth is unfavorable. Indeed, careful monitoring of either strain (directly) or of proxies for the dilatancy, such as enhanced fluid loss (permeability increase) could be integrated into operational procedures such as the traffic light system^6^ to avoid the largest induced seismic events, typically caused by the rapid propagation of large fractures at high rate. Previous studies have shown the potential for cyclic pressurization to induce staged fracture growth^15^ and develop fracture patterns dominated by interacting small fractures^7^^,^^9^^,^^30^ which are ideal for sustaining permeability to prolong energy extraction.^17^ Furthermore, modeling of rock masses subjected to cyclic pressure pulsing has shown that fracture network complexity increased with increasing number of loading cycles.^31^ Thus, cyclic pressurization, with integrated monitoring of induced dilatancy could be used to sequentially increase permeability and maximize potential of a geothermal site.

In conclusion, although here we test a new square wave and systematically control cyclic parameters, we show that our results agree broadly with cyclic and fatigue tests in a range of stressing geometries and scenarios^19^ (Figure 4). This speaks to the universality of the impact of cyclicity and fatigue in geomaterials, even when applied by fluid overpressure. We anticipate that this agreement between our results and results from other stress fields and lithologies therefore translates to the applicability of cyclic hydraulic stimulation in a broad spectrum of contexts including both hard rock and porous-permeable rock, and even those with pre-existing fractures. As such, we stress the need to optimize cyclic hydraulic pressure pulse strategies to maximize the extraction of geoenergy resources whilst minimizing the risk of large, unchecked fracturing events and reducing peak power consumption during geothermal exploitation.

### Limitations of the study

Following the initiation of a hydraulic fracture in our cyclic pressurization tests the fracture proceeded to grow rapidly and reach both sides of the sample in all cases as the pressure continued to be delivered due to the pressurized bladder accumulator, and the relatively small volume of fluid in the experimental sample. Unfortunately, this experimental artifact meant the fracture grew beyond the first initiation, and prevented the comparison of fracture geometry between the monotonic and cyclic cases. In a previous series of tests on PMMA samples, the same applied square waves without the bladder accumulator induced staged fracture growth^15^ which we also expect would be the case in geomaterials, based on other studies such as Zhou et al.,^30^ though we cannot assert this conclusively.

## Resource availability

### Lead contact

Further information and requests should be directed to and will be fulfilled by the lead contact, Jackie Evan Kendrick.

### Materials availability

The rock samples used for the experiments are stored at the University of Edinburgh. Off-cuts made when coring the experimental specimens were discarded. No other materials were used or produced.

### Data and code availability

• All data are either directly available in the article and supplement, or for the pressure data publicly available as of the date of publication on Github: https://github.com/Jackie-Kendrick/Kendrick-et-al.-cyclic-hydraulic-pressure-.git In all files, the first column is Relative time in [s]; the second column is Fluid pressure in [MPa]. No original code was used for the processing or plotting of data in this article. Any additional information required to reanalyze the data reported in this article is available from the lead contact upon request.

## Acknowledgments

This work was funded by the 10.13039/100014013UK Research and Innovation (10.13039/100014013UKRI) 10.13039/501100000266Engineering and Physical Sciences Research Council (10.13039/501100000266EPSRC) grant no. EP/S005560/1. For the purpose of open access, the authors have applied a Creative Commons Attribution (CC BY) licence (where permitted by 10.13039/100014013UKRI, “Open Government Licence” or “Creative Commons Attribution No-derivatives (CC BY-ND) licence” may be stated instead) to any Author Accepted Manuscript version arising. We thank Silixa for their assistance with the optical fibers.

## Author contributions

JEK, JM-C, AF-H, KE, CIMD, and ZKS devised the experimental program. JEK, JM-C, AF-H, and ALi designed the experimental set-up. JEK and JM-C conducted the experiments. ALi prepared the samples. JEK, ALa, and AF-H processed the data and produced the figures. JEK produced the first draft of the article and all authors contributed to editing it.

## Declaration of interests

The authors declare no competing interests.

## STAR★Methods

### Key resources table

**Table:** 

REAGENT or RESOURCE	SOURCE	IDENTIFIER
**Deposited data**		

Pressure data	This paper	https://github.com/Jackie-Kendrick/Kendrick-et-al.-cyclic-hydraulic-pressure-.git

**Software and algorithms**		

MatLab	MatLab 2024a	https://de.mathworks.com/products/new_products/latest_features.html
LabVIEW	LabVIEW NXG 3.0	https://www.ni.com/de.html

**Other**		

G603 Granite	Not applicable	https://wap.stonecontact.com/g603-granite/s2867

### Method details

#### Sample materials and preparation

The samples used are construction grade granite G603. Petrographic descriptions of the mineral assemblage and grain size were made from saw-cut surfaces. No petrophysical data are collected herein as our previous study (Kendrick et al.^22^) used the offcuts of the sample blocks used here to measure porosity by helium pycnometry and the uniaxial compressive and Brazilian tensile strengths. We quote these values herein as directly relevant to our tested materials and refer the reader to Kendrick et al.^22^ for more details.

Blocks of the G603 granite were cored to 200 mm lengths, and then cut and ground plane parallel to a length of 200 mm. A borehole of 8 mm diameter was drilled to a depth of 100 mm in the centre of the top surface of each sample. The sample size chosen represents a minimum dimension of at least 10 times the maximum length of the largest heterogeneity in the samples as defined by ASTM standard D7012.^32^

#### Radial strain

A fiberre optic strain cable was fitted around the circumference of the sample, providing high density strain measurements (every 2.6 mm). The fibre was bonded to the sample using Loctite® Super Glue. Optical fibre strain measurements were logged via a LUNA module and the ODISI-B acquisition system at 25 Hz frequency.^33^ The strain was zeroed after placing the sample into the uniaxial press and securing with contact load, and prior to applying the experimental axial stress. During logging the ODISI-B acquisition system logs the strain at each position, but, if the accuracy of the measurement drops below the defined quality factor the value NaN is recorded and no data are seen. In some cases NaN values are recorded when large displacements occur between successive measurements.

#### Experimental setup

The experimental set-up includes the sample and attached sensors, a fluid pressure delivery system and a uniaxial press to apply axial load and affix the platens to the sample. Before the sample was placed into the sample assembly, we primed the borehole to extract any air bubbles. To deliver the pressure pulses to the central borehole the sample assembly was loaded into a uniaxial press. The fluid pressure was delivered to the sample via a hollow 200 mm diameter platen with BS113 nitrile o-ring seal around the fluid entry port. Initially, an axial load of ∼0.1 MPa was applied, before extracting any further trapped air from the sample and pressure line by running the pump at low rate with the valve between pump and sample open, and an overflow valve at the platen entry also open. Once all air was evacuated the sample was isolated. Before proceeding with the tests an axial stress of 8 MPa was applied to the sample using an Enerpac automated pump which held a constant hydraulic fluid pressure on the axial load-string, logged on the display at a frequency of 1 Hz.

#### Monotonic tests

Two monotonic hydraulic fracturing tests were conducted under an axial stress of 8 MPa to determine the breakdown pressure with a non-pulsed fluid applied at constant flow rate. A Teledyne Isco - 100DX Syringe Pump was used to supply a constant flow rate of 1 ml.min^-1^ so that the fluid pressure rose non-linearly until sample failure. At the laboratory temperature of about 21°C water has a viscosity of around 1.0 mPa.s. The average breakdown pressure from the two tests was used to define the conditions of cyclic tests to be conducted.

#### Cyclic tests

A fluid pressure delivery system was developed to deliver square cyclic pressure pulses (square waves) of water to the central borehole of the samples see Mouli-Castillo et al.^15^ The set-up uses a Cole-Parmer Constant-Flow Dual Piston Pump attached to parallel low and high-pressure fluid lines. A solenoid valve controlled the shift from a high-to low-pressure line at controlled intervals.

The target pressures were set in the high- and low-pressure lines using back-pressure regulators bleeding off excess fluid, with fluid pressure maintained by supplying a constant flow rate and buffered by a gas-filled pressure regulator (bladder accumulator) set to the pressure of the high-pressure line to smooth the pressure pulses. This set-up allowed the pump to function under stable conditions at a range of high- and low-pressure line settings. Pressures were chosen as a fraction of the monotonic breakdown pressure according to our experimental program shown in Table 1.

A LabVIEW program controlled the assembly and switched from high to low pressure every 3.57 s, a cycle frequency of 0.14 Hz. The LabVIEW controller recorded fluid pressure at the inlet of the sample at a rate of 500 Hz to ensure consistency of the pulse pressures. Only once the pressure conditions had stabilised, the samples exposed to the pressurised line by opening the valve between the fluid pressure line and the sample. The samples were then exposed to 3.57 s of high-fluid pressure, followed by 3.57 s of low-fluid pressure until failure.

The cyclic fluid pressurisation system was used to investigate the effect of square waves on the fatigue life of granite, extending the cyclic hydraulic fracture literature that has typically focused upon sinusoidal or pressure spiked pulses. In the experiments we controlled mean pressure (P_mean_) and cycle amplitude, hence also maximum (P_max_) and low (P_min_) and minimum pressure of the cycles. The rapid shifts in pressure we induced are equivalent to anticipated pressure changes in a geothermal borehole manipulated by valve shifting of a high and low pressure line.

## Supplemental information

Document S1. Figures S1–S11

Video S1. Video of the strain recorded before, during, and after sample failure in the monotonic test is shown in Figure 2 in the article

Video S2. Video of the strain recorded before, during, and after sample failure in the cyclic test is shown in Figure S4, with Pmax = 90%, Pmin = 60%, and Pmean = 75%

Video S3. Video of the strain recorded before, during, and after sample failure in the cyclic test is shown in Figure S5, with Pmax = 95%, Pmin = 55%, and Pmean = 75%

Video S4. Video of the strain recorded before, during, and after sample failure in the cyclic test is shown in Figure S6, with Pmax = 95%, Pmin = 65%, and Pmean = 80 % Note that in this sample the strain was not zeroed before the test so the signal is poor.

Video S5. Video of the strain recorded before, during, and after sample failure in the cyclic test is shown in Figure S7, with Pmax = 95%, Pmin = 75%, and Pmean = 85%

Video S6. Video of the strain recorded before, during and after sample failure in the cyclic test is shown in Figure S8, with Pmax = 85%, Pmin = 75%, and Pmean = 80%

Video S7. Video of the strain recorded before, during and after sample failure in the cyclic test shown in Figure 3, with Pmax = 95%, Pmin = 85% and Pmean = 90%

Video S8. Video of the strain recorded before, during, and after sample failure in the cyclic test is shown in Figure S9, with Pmax = 90%, Pmin = 80%, and Pmean = 85%

Video S9. Video of the strain recorded before, during and after sample failure in the cyclic test is shown in Figure S10, with Pmax = 85%, Pmin = 65%, and Pmean = 75%
