# Supplementary material for: Validating the application of cyclic hydraulic pressure pulses to reduce breakdown pressure in granite
Source: iScience. 2024 Sep 3;27(10):110881. doi: 10.1016/j.isci.2024.110881 (PMC11424943; doi:10.1016/j.isci.2024.110881)
Supplement: Document S1. Figures S1–S11 [file mmc1.pdf]

iScience, Volume *27*

Supplemental information

Validating the application of cyclic hydraulic pressure pulses to reduce breakdown pressure in granite

Jackie Evan Kendrick, Anthony Lamur, Julien Mouli-Castillo, Alexander Lightbody, Andrew Fraser-Harris, Katriona Edlmann, Christopher Ian McDermott, and Zoe Kai Shipton

Supplementary figures

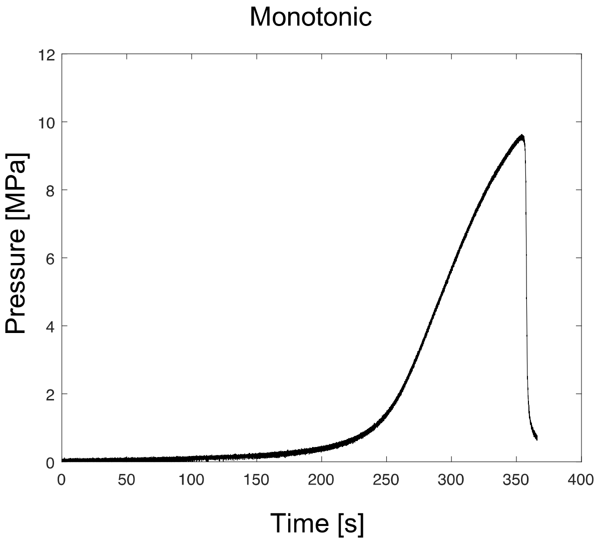

Supplementary Figure 1: Repeat monotonic test with constant flow rate.

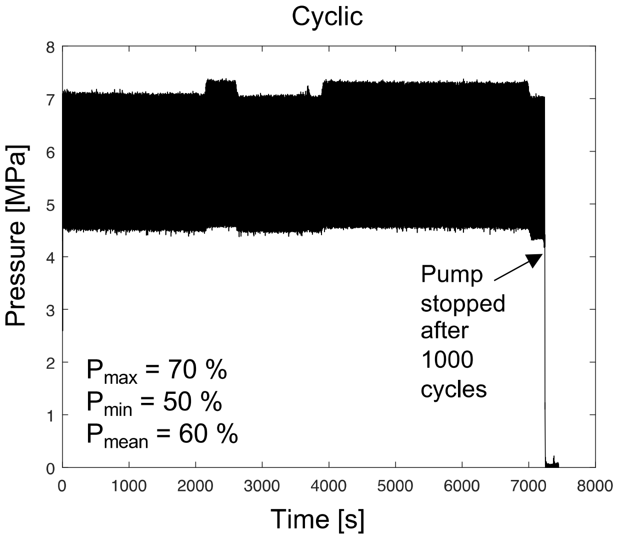

Supplementary Figure 2: Cyclic test in which the sample survived to 1000 cycles.

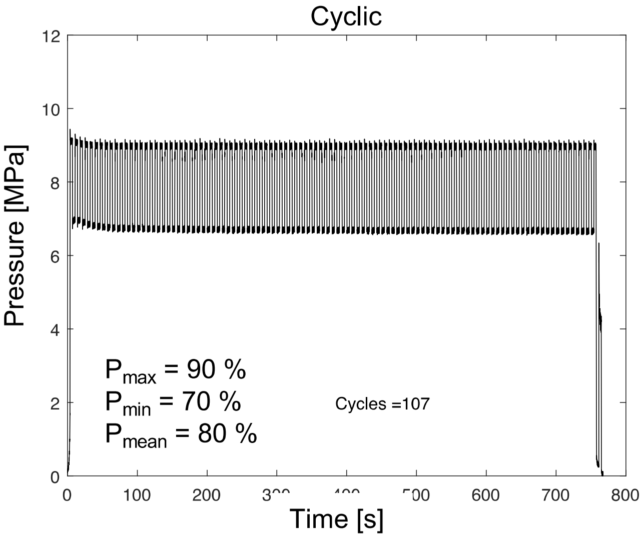

Supplementary Figure 3: Cyclic test in which the sample failed at 107 cycles.

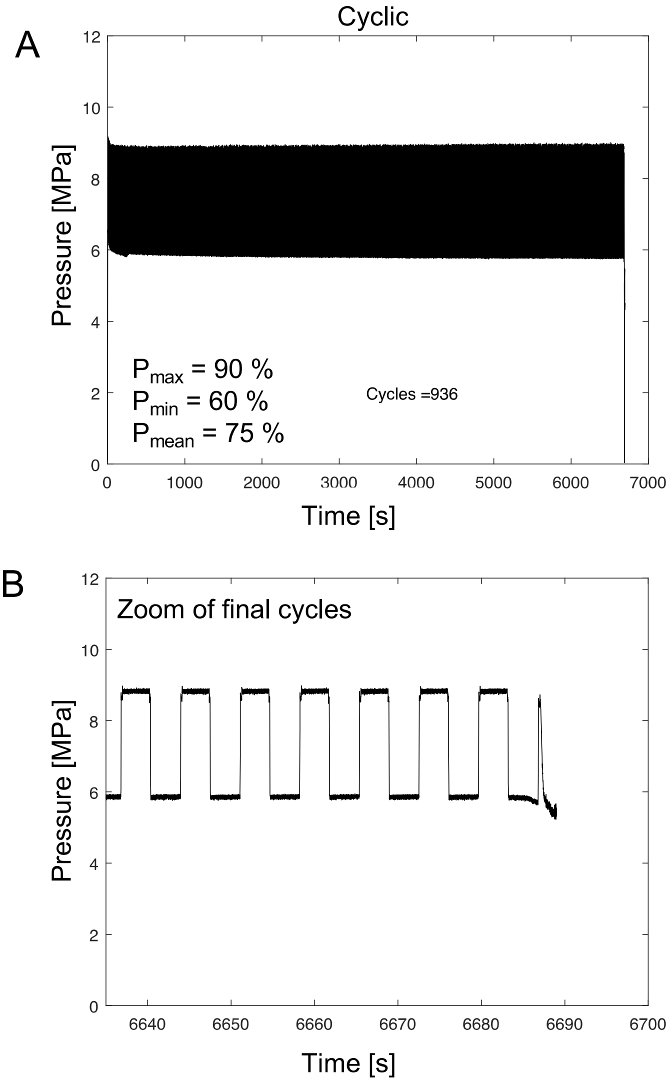

Supplementary Figure 4: Cyclic test in which the sample failed at 936 cycles, (A) whole test and (B) the final cycles leading to failure.

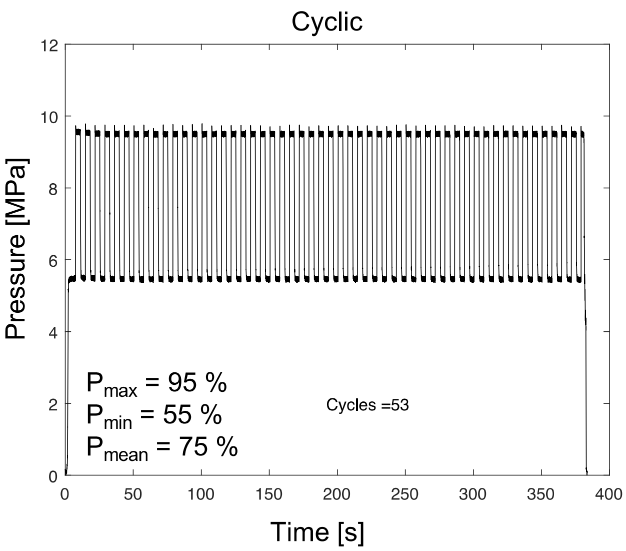

Supplementary Figure 5: Cyclic test in which the sample failed at 53 cycles.

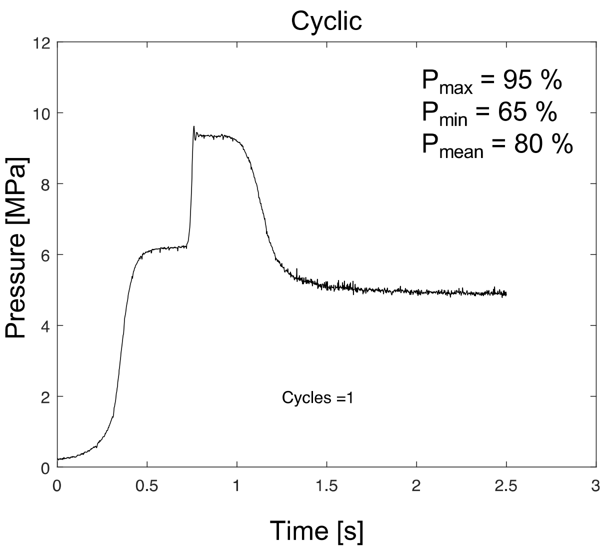

Supplementary Figure 6: Cyclic test in which the sample failed on the first cycle.

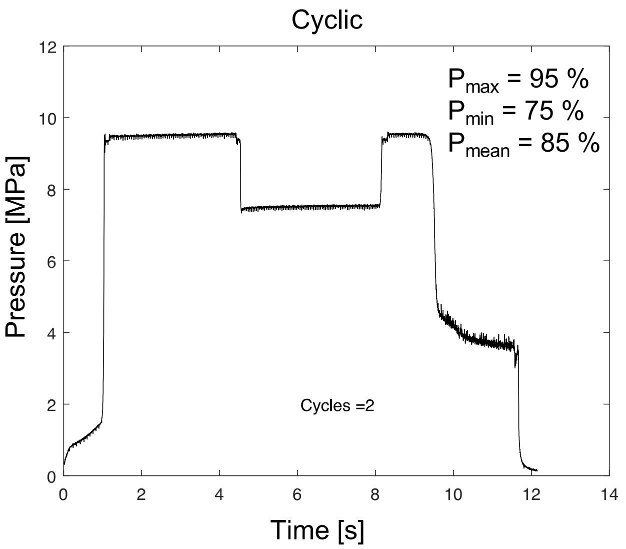

Supplementary Figure 7: Cyclic test in which the sample failed at 2 cycles.

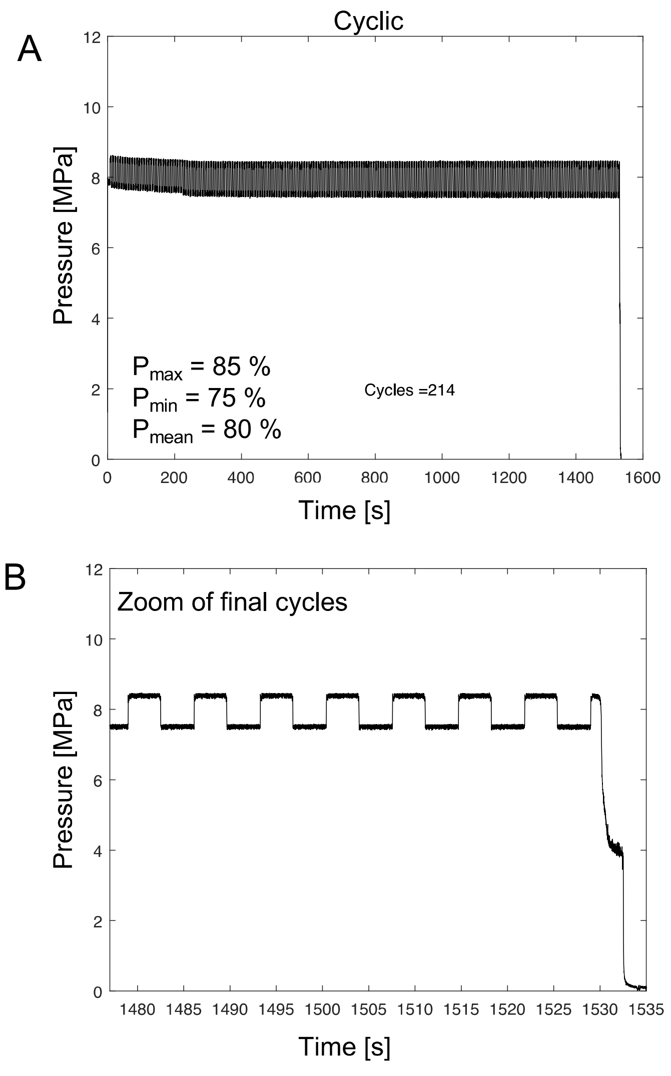

Supplementary Figure 8: Cyclic test in which the sample failed at 214 cycles, (A) whole test and (B) the final cycles leading to failure.

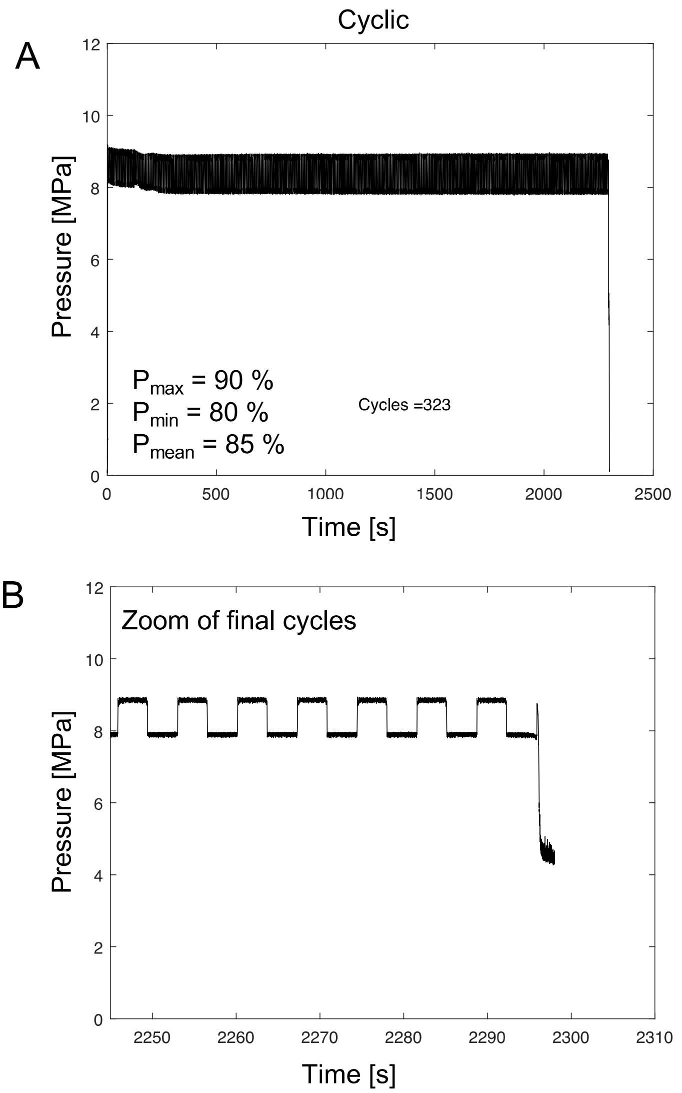

Supplementary Figure 9: Cyclic test in which the sample failed at 323 cycles, (A) whole test and (B) the final cycles leading to failure.

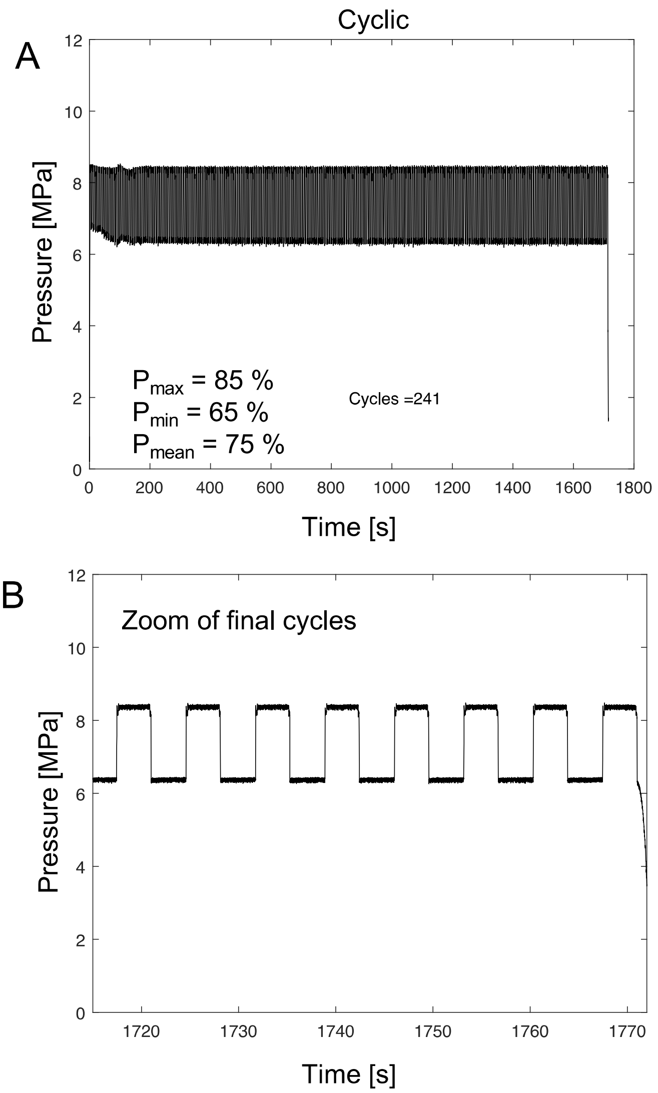

Supplementary Figure 10: Cyclic test in which the sample failed at 241 cycles, (A) whole test and (B) the final cycles leading to failure.

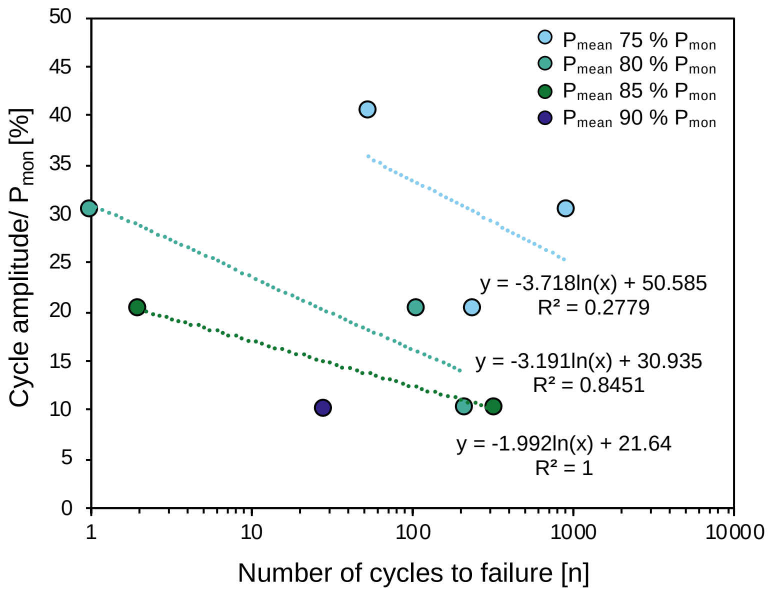

Supplementary Figure 11: Regressions fitted to each pressure pulsing dataset, used to define the exponents (-3.718, -3.191 and -1.992 respectively for P_{mean} = 75 %, 80 % and 85 % of P_{mon}) which were then averaged to create a regression with exponent -2.96 (S.D. 0.88), depicted by the contours in Figure 5.
